# Patterns and drivers of benthic macroinvertebrate assemblages in the kelp forests of southern Patagonia

**DOI:** 10.1371/journal.pone.0279200

**Published:** 2023-01-06

**Authors:** Alan M. Friedlander, Enric Ballesteros, Jennifer E. Caselle, Mathias Hüne, Alyssa M. Adler, Enric Sala

**Affiliations:** 1 Pristine Seas, National Geographic Society, Washington, DC, United States of America; 2 Hawaiʿi Institute of Marine Biology, University of Hawaiʿi, Kāneʻohe, Hawaiʿi, United States of America; 3 Centre d’Estudis Avançats de Blanes-CSIC, Blanes, Girona, Spain; 4 Marine Science Institute, University of California Santa Barbara, Santa Barbara, California, United States of America; 5 Centro de Investigación para la Conservación de los Ecosistemas Australes (ICEA), Punta Arenas, Chile; 6 Division of Marine Science and Conservation, Nicholas School of the Environment, Duke University, Beaufort, North Carolina, United States of America; Universita degli Studi di Genova, ITALY

## Abstract

The kelp forests of southern Patagonia have a large diversity of habitats, with remote islands, archipelagos, peninsulas, gulfs, channels, and fjords, which are comprised of a mixture of species with temperate and sub-Antarctic distributions, creating a unique ecosystem that is among the least impacted on Earth. We investigated the distribution, diversity, and abundance of marine macroinvertebrate assemblages from the kelp forests of southern Patagonia over a large spatial scale and examined the environmental drivers contributing to the observed patterns in assemblage composition. We analyzed data from 120 quantitative underwater transects (25 x 2 m) conducted within kelp forests in the southern Patagonian fjords in the Kawésqar National Reserve (KNR), the remote Cape Horn (CH) and Diego Ramírez (DR) archipelagos of southern Chile, and the Mitre Peninsula (MP) and Isla de los Estados (IE) in the southern tip of Argentina. We observed rich assemblages of macroinvertebrates among these kelp forests, with a total of 185 unique taxa from 10 phyla and 23 classes/infraorders across the five regions. The number of taxa per transect was highest at IE, followed by MP, CH, and KNR, with the lowest number recorded at DR. The trophic structure of the macroinvertebrate assemblages was explained mostly by wave exposure (28% of the variation), followed by salinity (12%) and the KNR region (11%). KNR was most distinct from the other regions with a greater abundance of deposit feeders, likely driven by low salinity along with high turbidity and nutrients from terrigenous sources and glacial melt. Our study provides the first broad-scale description of the benthic assemblages associated with kelp forests in this vast and little-studied region and helps to establish baselines for an area that is currently lightly influenced by local anthropogenic factors and less impacted by climate change compared with other kelp forests globally.

## Introduction

The marine ecosystems of southern Patagonia are among the least impacted on the planet. The region is geomorphologically complex, with archipelagos, peninsulas, gulfs, channels, and fjords, which have been shaped by ice expansion and contraction during the Quaternary glacial period, giving the area high heterogeneity and diversity of nearshore habitats [1–3]. Habitat heterogeneity is also influenced by freshwater discharge from the melting of four large ice fields (Southern Patagonia, Muñoz-Gamero Península, Santa Inés Island, and Cordillera Darwin), producing high environmental variability, with a strong salinity gradient between glacial fjords and islands and coasts exposed to oceanic conditions [4, 5]. The confluence of water masses from the Pacific, Atlantic, and Southern oceans, which mix through the Drake Passage, straits of Le Maire and Magellan, and the Beagle Channel result in highly diverse marine communities with a mixture of species of temperate and sub-Antarctic distributions [6, 7]. This diversity of oceanographic, geomorphologic, and environmental characteristics creates a unique ecosystem at the southern tip of South America.

Kelps are structurally complex and highly productive habitat builders of cold-water rocky marine coastlines [8–12]. In southern Patagonia, they dominate the nearshore marine community. These sub-polar and temperate algal forests are found along a high gradient of geographical coastal diversity, from the protected fjords of southern Chile to the exposed shorelines of Cape Horn and Diego Ramírez islands [13–15]. These highly varied habitats are complemented by a variety of substrate types and fluctuating freshwater influences. Despite wide-ranging variance in environmental conditions, the giant kelp *Macrocystis pyrifera* plays a key and persistent role in structuring the ecological communities of the entire region. The large brown kelp *Lessonia* spp. usually forms dense understory within these *Macrocystis* forests. Additional species of brown, red, and green algae colonize the seafloor in a heterogenous mix.

In Patagonia, kelp forests have rich and diverse benthic invertebrate assemblages [13, 14, 16–18]. Kelp structure provides shelter, while also buffering wave and current energy for other seafloor fauna and flora [12, 19]. In return, herbivores and mesopredators in this ecosystem maintain kelp forest conditions by removing epiphytes from brown algae and reducing competition for space by grazing turf algae from substrate suitable for kelp recruitment [20–22]. In the Northeast Pacific, this balance is achieved by strong top-down control by predators [23, 24]. However, in Patagonia and the South Atlantic, a parallel mechanism has not been described.

The kelp forests of southern South America host recreationally and commercially important invertebrate species through a variety of life stages [13, 19]. Examples include the Patagonian long-fin squid *Loligo gahi*, which selectively lay their egg clutches on *Macrocystis pyrifera* or *Lessonia* spp. near the forest edge, and both juvenile king crab *Lithodes santolla* and false king crab *Paralomis granulosa*, which have been observed seasonally in large aggregations within southern Chile’s *Macrocystis* forests [25–30]. Commercial fisheries for these species throughout Patagonia and the South Atlantic provide economic opportunities for regional coastal communities. Owing to the remoteness of this region, much is still to be learned about the various life stages and habitat requirements of many species inhabiting these kelp forests, especially those species that are not currently exploited.

Global stressors have recently impacted kelp forests, particularly in the past decade [31–34]. Many of these impacts directly or indirectly (through the loss of kelp structure) affect the invertebrate communities that rely on forest structure [34–36] and can cause collapse of fisheries where those invertebrate species are fished. Ocean acidification weakens the calcium carbonate shell of many animals in their larval stage, reducing viability and recruitment [37, 38]. Ocean warming encourages movement of invasive species into habitats that are not protected against novel immigrants [39, 40]. An increase in surface seawater temperature results in a strong thermocline, which acts as a buffer between less productive surface water and nutrient-rich deep water. This thermocline reduces mixing potential between layers and prevents important nutrients that brown algae like kelp rely on from reaching nearshore systems [41].

Invertebrate assemblages have previously been investigated in kelp forest habitats of Patagonia [16, 42], but no study has explored how environmental conditions may impact these important communities on a large spatial scale. The data used here were collected during the austral summer of 2017, 2018, and 2020 as part of several National Geographic Pristine Seas expeditions. We characterized the invertebrate community associated with *Macrocystis pyrifera* and *Lessonia* spp. forests across three distinct regions: (1) Kawésqar National Reserve (KNR) in the fjords adjacent to the Straits of Magellan, characterized by channel and fjord ecosystems; (2) Isla de los Estados (IE) and Mitre Peninsula (MP) at the easternmost extent of Tierra del Fuego, Argentina; and (3) the Cape Horn (CP) and Diego Ramírez (DR) archipelagos, at the southernmost tip of South America.

The objectives of this study were to: (1) assess the spatial patterns in kelp forest benthic community structure, and compare invertebrate assemblages between areas, (2) determine which biotic and abiotic factors influence diversity and abundance, and (3) provide a baseline of the spatial distribution of invertebrate assemblages for this remote region so future changes in community structure can be assessed.

## Material and methods

### Ethics statement

Data were collected by all authors in a collaborative effort. Non-invasive research was conducted, which included photographs, and visual estimates described in the methods below. The Republic of Argentina and the Republic of Chile granted all necessary permissions to conduct this research. No vertebrate sampling was conducted and therefore no approval was required by any Animal Care and Use Committee. Our data are publicly available at Data Dryad: doi.org/10.5061/dryad.931zcrjpx.

### Study area

**Fig 1:**
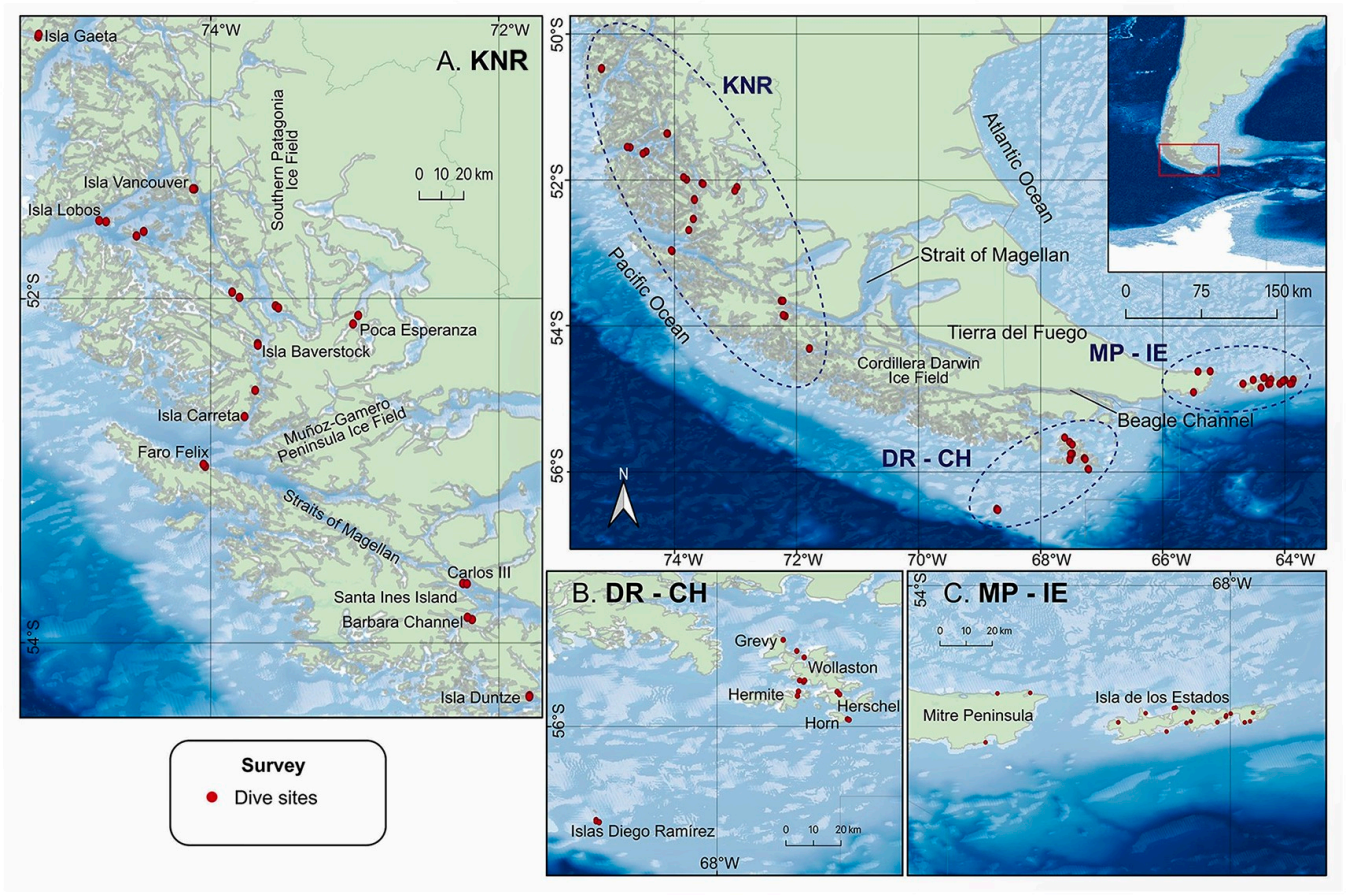
Sampling stations in southern Patagonia. A. KNR = Kawésqar National Reserve, B. DR = Diego Ramírez and CH = Cape Horn Archipelago, C. MP = Mitre Peninsula and IE = Isla de los Estados. Basemap derived from GEBCO Compilation Group (2020) GEBCO 2020 Grid (doi:10.5285/a29c5465-b138-234de053-6c86abc040b9). Processing and assembly of the Global Self-consistent, Hierarchical, High-resolution Geography Database for shoreline data from [44].

**Fig 2:**
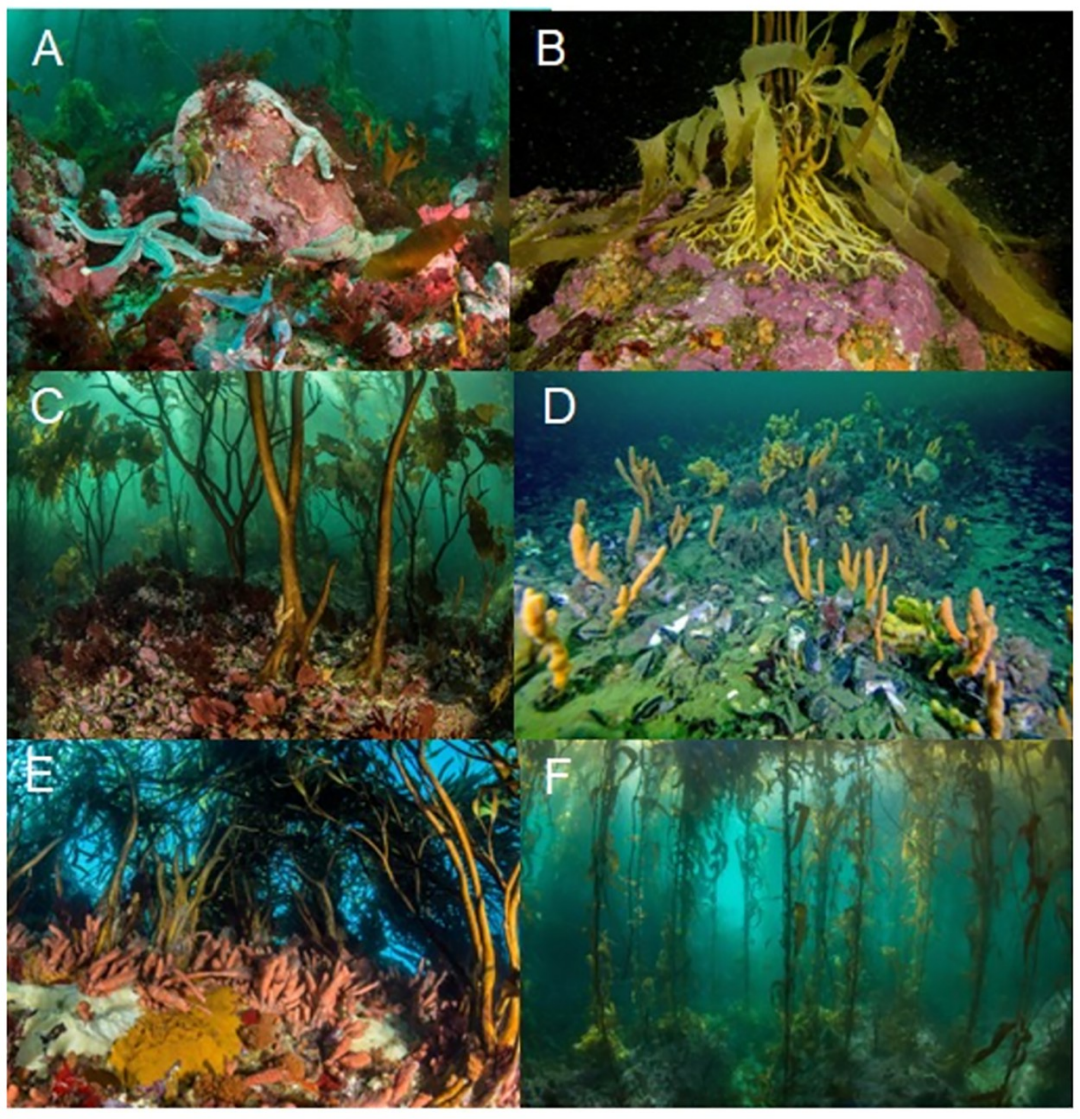
Representative marine benthic habitats of southern Patagonia. A. Cape Horn, B. Mitre Peninsula, Argentina, C. Isla de los Estados, Argentina, D. Kawésqar National Reserve, Chile, E. and F. Diego Ramírez, Chile.

Surveys were conducted within shallow forests of giant kelp, *Macrocystis pyrifera* (4–18 m depth), in five areas (Kawésqar National Reserve—KNR, Isla de los Estados—IE, Mitre Peninsula—MP, Cape Horn—CH, Diego Ramírez—DR) along approximately 800 km of the southern coast of South America between Gaeta Island (50.48°S 75.19°W) to the north and DR (56.5°S 68.7°W) to the south, and IE (54.7°S 64.5°W) in the southeast (Fig 1). The kelp forests across this vast region comprise a wide range of diversities and complexities (Fig 2).

Cape Horn is the southernmost headland of the Tierra del Fuego Archipelago, marking the northern boundary of the Drake Passage where three great oceans meet [43]. The Diego Ramírez Islands are a small archipelago located on the southern edge of the continental shelf ca. 105 km west-southwest of Cape Horn and ca. 700 km northwest of the South Shetland Islands and the Antarctic Peninsula. The Kawésqar National Park and its adjacent marine National Reserve (KNR) consists of a diverse range of terrestrial and marine habitats that includes the southern terminus of the Andes, the Southern Patagonia Ice Fields, sub-Antarctic rainforests, glaciers, fjords, lakes, wetlands, valleys, channels, and islands. The Mitre Peninsula (MP) is the easternmost extent of Isla Grande de Tierra del Fuego, with Isla de los Estados (IE) lying 29 km to the east and separated by the Le Maire Strait. Shorelines directly exposed to predominant ocean swells were classified as exposed, semi-closed channels and ocean facing fjords were classified as semi-exposed, and fjords and inland bays protected from westerly winds and swells were classified as sheltered.

### Survey method

Characterization of the macrobenthos was conducted by scuba divers along two 25-m long transects at each sampling location. Transects were run parallel to the shoreline, with a target depth of 10 m, depending on location of the lower edge of the kelp forest. Since sampling sites targeted kelp forests only, the underlying habitats were biased towards hard substrate.

For sessile and mobile invertebrates, the number of individuals was estimated on 1-m of either side of the transect line (50 m^2^). For colonial organisms (sponges, some cnidarians, bryozoans, and some tunicates) colonies, rather than individuals, were counted. When a species was extremely abundant (i.e., > 500) along the transect, abundance was estimated considering the number of individuals/colonies m^-2^ and scaled to the total area of the transect (50 m^2^). Only non-cryptic invertebrates ≥1 cm were enumerated. A second diver counted the number of kelp (*Macrocystis pyrifera* and *Lessonia* spp.) stipes within 1-m on either side of the transect and recorded the bottom type for each transect. Salinity and temperature measures were recorded at each site using a YSI model 556 handheld multiparameter instrument or RBR concerto multi-channel logger. Salinity was recorded in parts per thousand (ppt) ± 0.1 and temperature was recorded in °C ± 0.1.

### Statistical analyses

Comparisons of abiotic (temperature, salinity, depth) and biotic metrics (densities of *Macrocystis pyrifera* and *Lessonia* spp. per transect), as well as kelp forest community metrics (taxa richness, numerical abundance, diversity, and evenness) among regions were conducted using Generalized Liner Models with Poisson distributions and log-link functions. Pair-wise comparisons between regions were performed with contrasts using -Loglikelihood estimates (α = 0.05).

Benthic taxonomic diversity was calculated using the Shannon-Wiener diversity index [45]: H´ = -Ʃ p_i_ ln(p_i_), where *p*_*i*_ is the proportion of all individuals counted of taxa *i*. The evenness component of diversity was expressed as: J = H´/ln(S), where S is the total number of species present. Benthic taxa were categorized by functional (trophic) groups based on published literature and were as follows: passive suspension feeder, active suspension feeder, herbivorous/browser, carnivorous, omnivorous, and deposit feeder. An index of relative dominance (IRD) for each taxa was created by multiplying the percent frequency of occurrence of the taxa on each transect by the relative percent numerical abundance of that taxa x 100 [46].

To describe the pattern of benthic community structure among regions and their relationship to biotic and abiotic variables, direct gradient analysis (redundancy analysis, RDA) was performed using the ordination program CANOCO version 5.0 [47]. The response variables were centered and ln(x+1) transformed benthic taxa numerical abundance data by region. Explanatory variables consisted of depth, salinity, temperature, latitude, longitude, habitat type, densities of *Macrocystis* and *Lessonia* kelp. All explanatory variables were centered and standardized prior to analysis. Pearson’s product-moment correlation analysis was used to test for dependence between explanatory variables. The variance inflation factor (VIF) of each explanatory variable measured the extent of multiple correlation with the other predictors in the RDA. Variables with large VIFs (> 20) do not bring any unique contribution to the explanatory power of the model. Latitude and longitude had high VIFs and were excluded from the analyses. To rank explanatory variables in their relevance to benthic community structure, interactive forward selection was used; the statistical significance of each variable was judged by a Monte Carlo unrestricted permutation test with 499 permutations [48].

## Results

### Regional characteristics

Temperature was significantly higher at KNR compared to the other regions and intermediate at IE (Table 1). Salinity was significantly lower in KNR compared to the other regions and had the highest variance (COV = 15.44%). Transect depth, which was approximately equivalent to the seaward extent of the kelp forest, was significantly shallower in KNR compared to the insular regions. Transect depth at MP was intermediate to KNR and the insular regions. Densities of *Lessonia* spp. were significantly higher at DR than all other regions, with CH and IE significantly higher than the two continental regions of MP and KNR. Densities of *Macrocystis* were not significantly different between MP, IE, and DR but were all higher than CH and KNR, which were not significantly different from one another.

**Table 1:** Abiotic and biotic comparisons among regions in southern Patagonia. Results of Generalized Linear Models with Poisson distributions and log-link functions. Values among regions are means and one standard deviation. Pair-wise comparisons between regions were performed with contrasts using -Loglikelihood estimates. Regions with the same letter are not significant different (α = 0.05). Region codes are described in the text and Fig 1.

Metric	-LogLikelihood	χ^2^	p		
Temp. (°C)	97.04	194.08	<0.001		
Salinity (ppt)	79.12	158.25	<0.001		
Depth (m)	68.44	136.88	<0.001		
*Lessonia*	36.96	73.91	<0.001		
*Macrocystis*	15.98	31.96	<0.001		
Metric	CH	DR	IE	KNR	MP
Temp. (°C)	9.5 (0.6)	9.5 (0.6)	8.0 (0.5)	10.5 (1.2)	8.8 (1.1)
Contrasts	C	C	B	A	C
Salinity (ppt)	32.9 (0.1)	33.5 (0.1)	32.8 (0.3)	26.2 (4.1)	33.2 (0.5)
Contrasts	AB	A	A	B	A
Depth (m)	12.8 (1.9)	13.5 (1.2)	12.9 (2.9)	7.5 (2.1)	10.4 (5.9)
Contrasts	AB	A	AB	C	B
*Lessonia*	1.2 (1.5)	3.5 (1.7)	1.4 (1.4)	0.3 (0.4)	0.3 (0.4)
Contrasts	B	A	B	C	C
*Macrocystis*	4.2 (2.9)	6.4 (2.7)	6.5 (2.9)	3.7 (2.0)	7.1 (2.4)
Contrasts	B	A	A	B	A

### Invertebrate assemblages

We observed a total of 185 unique taxa from 10 phyla and 23 class/infraorder among the five regions (S1 Table). The most taxa-rich phyla were Mollusca (n = 43), followed by Echinodermata (n = 33), Porifera (n = 24), Cnidaria (n = 20), Chordata (n = 19), Ectoprocta (n = 18), and Arthropoda (n = 18). The most dominant species based on IRD was *Balanus laevis*, which accounted for 15.2% of total abundance and occurred in 41.7% of all transects (Table 2). This was followed by the bivalve *Aulacomya atra*, which occurred in 27.5% of the transects and comprised 14.2% of total abundance. The bivalve *Gaimardia trapesina* was the most numerically dominant species but only occurred in 18.3% of the transects. The ascidian *Didemnum studeri* only accounted for ~ 3% of total numerical abundance but occurred on 85.0% of all transects.

**Table 2:** Top 15 benthic taxa overall among all transects (n = 120). Taxa are ordered by index of relative dominance (IRD) = % frequency of occurrence (Freq. %) x % numerical abundance (Abund. %).

Phyla	Class to Infraorder	Taxa	Num. abun. (sd)	Freq. %	Abund. %	IRD
Arthropoda	Cirripedia	*Balanus laevis*	6.38 (22.74)	41.67	15.22	634.23
Mollusca	Bivalvia	*Aulacomya atra*	5.96 (26.00)	27.50	14.21	390.78
Mollusca	Bivalvia	*Gaimardia trapesina*	8.76 (38.01)	18.33	20.90	383.20
Chordata	Ascidiacea	*Didemnum studeri*	1.24 (3.21)	85.00	2.97	252.07
Arthropoda	Malacostraca	*Pagurus comptus*	0.73 (1.98)	77.50	1.74	135.00
Echinodermata	Echinoidea	*Arbacia dufresnii*	0.73 (3.34)	72.50	1.75	126.87
Echinodermata	Echinoidea	*Loxechinus albus*	0.60 (1.22)	67.50	1.44	97.21
Echinodermata	Asteroidea	*Cosmasterias lurida*	0.43 (0.57)	88.33	1.02	90.31
Mollusca	Bivalvia	*Mytilus chilensis*	2.42 (17.36)	15.00	5.76	86.44
Echinodermata	Ophiuroidea	*Ophiactis asperula*	0.84 (2.39)	39.17	2.01	78.78
Mollusca	Gastropoda	*Nacella flammea*	0.36 (0.76)	79.17	0.85	67.09
Ectoprocta	Gymnolaemata	Unid. encrusting orange bryozoan	0.96 (5.89)	26.67	2.28	60.82
Echinodermata	Echinoidea	*Pseudechinus magellanicus*	0.33 (0.60)	69.17	0.79	54.94
Chordata	Ascidiacea	*Sycozoa gaimardi*	0.33 (1.05)	64.17	0.79	50.81
Mollusca	Gastropoda	*Margarella violacea*	0.27 (0.51)	60.83	0.64	38.64

**Fig 3:**
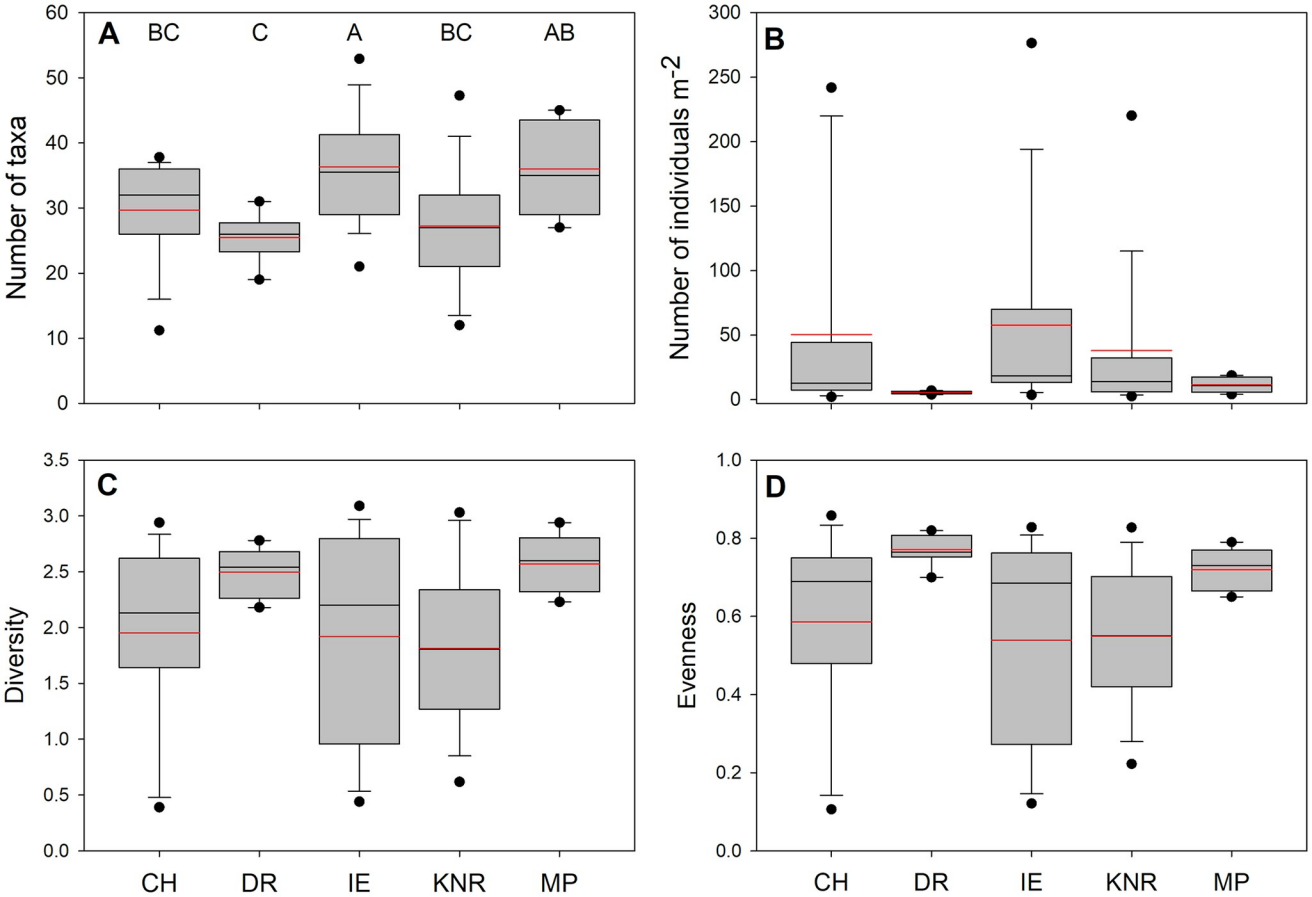
Comparisons of kelp forest community metrics among regions. A. Taxa richness, B. Numerical abundance (Num. individuals m^-2^), C. Shannon-Wiener Diversity, D. Pielou’s Evenness. Boxes represent 25th, median, and 75th percentiles, and upper and lower quartiles. Black horizontal lines are median and red horizontal lines are mean values. Regions with the same letter are not significantly different (α = 0.05). Results of Generalized Linear Models with Poisson distributions and log-link functions and pair-wise contrasts using -Loglikelihood estimates.

There was a significant difference in the number of taxa observed on transects among regions (χ^2^–25.65, p < 0.001; Fig 3). The number of taxa per transect was highest at IE, followed by MP, with the lowest number of taxa recorded at DR. There were no significant differences in the number of individuals m^-2^, diversity, or evenness among regions.

**Fig 4:**
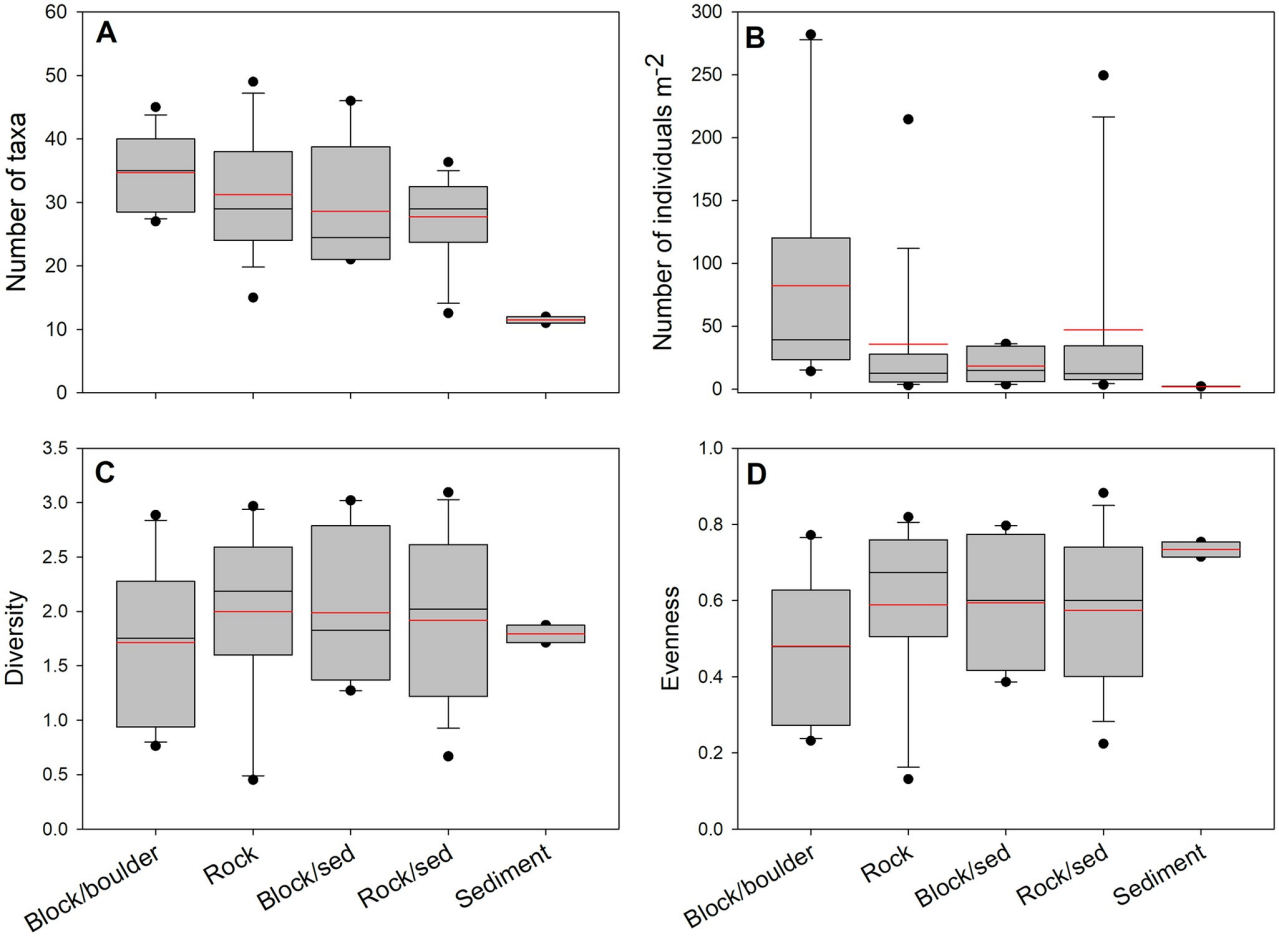
Comparisons of kelp forest community metrics among habitats. A. Taxa richness, B. Numerical abundance (Num. individuals m^-2^), C. Shannon-Wiener Diversity, D. Pielou’s Evenness. Boxes represent 25th, median, and 75th percentiles, and upper and lower quartiles. Black horizontal lines are median and red horizontal lines are mean values.

Since sampling sites targeted kelp forests, the underlying habitats were not pre-selected and skewed towards rock (55.8%), rock/sediment (25.0%), and block/boulder (10.8%), with block/sediment (6.7%) and sediment (1.7%) accounting for a small number of transects. This unbalanced design precluded statistical analyses; however, differences in invertebrate assemblage metrics were evident among habitat types. The number of taxa was highest in block/boulder habitat (34.7 ± 5.9), followed by rock (31.3 ± 9.9), block/sediment (28.6 ± 10.0), rock/sediment (27.7 ± 7.1), and sediment (11.5 ± 0.7) (Fig 4). The number of individuals m^-2^ was also highest in block/boulder habitat (82.3 ± 95.2), followed by rock/sediment (47.1 ± 77.4), rock (35.8 ± 59.2), block/sediment (18.5 ± 14.3), and sediment (2.1 ± 0.1). The differences in diversity and evenness were small and indistinct among habitats.

### Drivers of invertebrate assemblage structure

**Fig 5:**
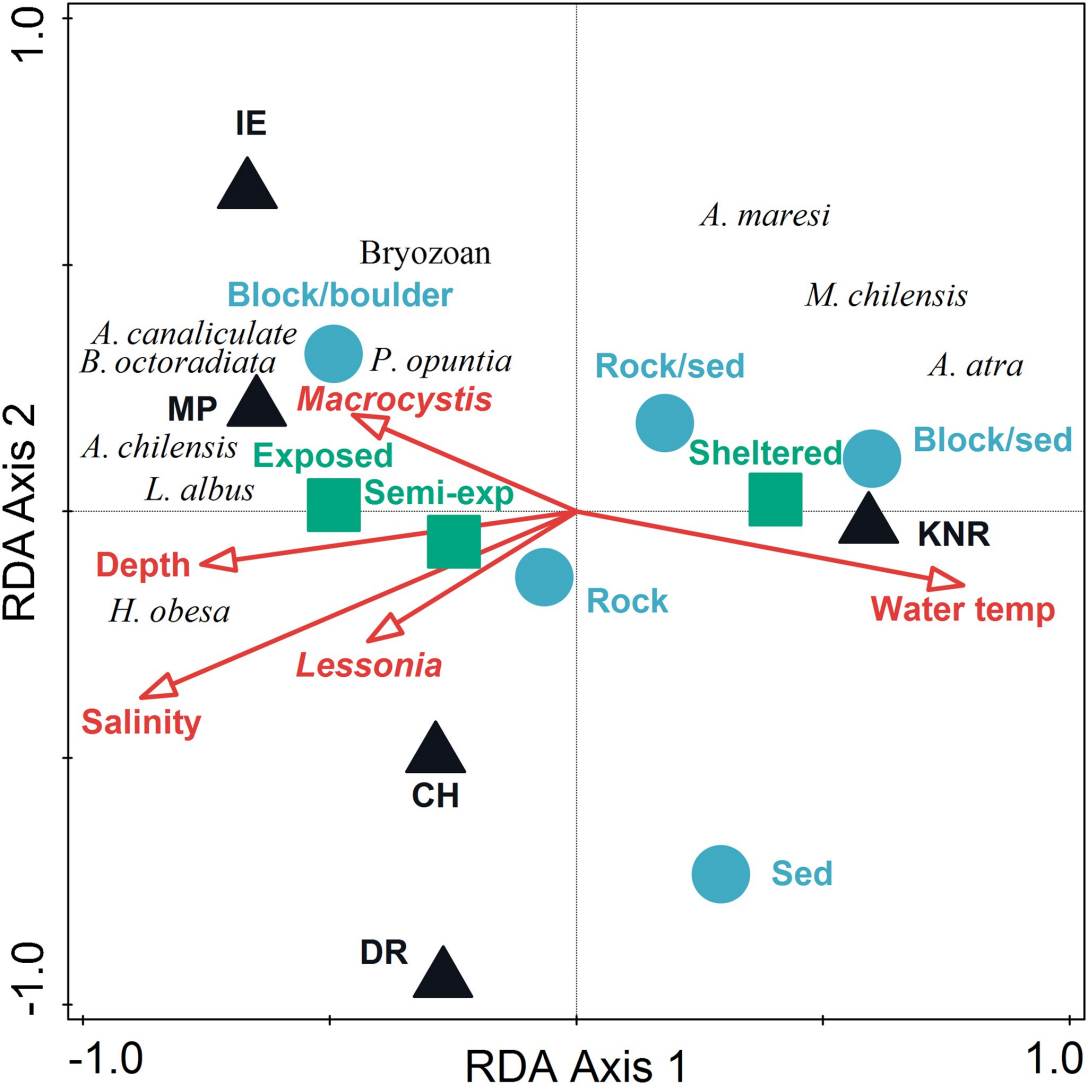
Triplot of results of redundancy analysis on benthic taxa abundance by region with biotic and abiotic variables (depth, salinity, temperature, habitat type, densities of *Macrocystis* and *Lessonia* kelp). Data were centered and ln(x+1) transformed benthic taxa abundance by transect. Biotic and abiotic variables were standardized prior to analysis. Taxa codes are as follows: *A*. *chilensis* = *Actinostola chilensis*, *A*. *atra* = *Aulacomya atra*, *A*. *maresi* = *Amphimedon maresi*, *A*. *canaliculata* = *Austrocidaris canaliculata*, *B*. *laevis* = *Balanus laevis*, *B*. *octoradiata* = *Bunodactis octoradiata*, *L*. *albus* = *Loxechinus albus*, *M*. *chilensis* = *Mytilus chilensis*.

Based on taxa abundance, regions were well separated from one another in ordination space (Fig 5). The first two axes of the RDA biplot explained 18.8% of the variance among benthic taxa, with the explanatory variables accounting for 49.4% of total variation (Table 3). RDA1 separated KNR from the other regions, while RDA2 separated CH and DR from the two sub-regions in Argentina (IE and MP). Salinity contributed 29.8% to the explained fitted variation, followed by IE (13.5%), DR (9.1%), and KNR (6.9%). All regions combined contributed 34.6% to the fitted variation, while all habitats combined accounted for 15.1%. All wave exposures combined comprised 12.9% of fitted variation.

**Table 3:** (a) Results of redundancy analysis (RDA) on ln(x+1) transformed benthic taxa abundance (num. m^-2^) by transect. (b) Conditional effects of Monte-Carlo permutation results on the RDA.

(a) Axes	Axis 1	Axis 2	Axis 3	Axis 4
Eigenvalues	0.134	0.054	0.042	0.030
Explained variation (cumulative)	13.380	18.800	22.950	25.970
Pseudo-canonical correlation	0.940	0.866	0.858	0.803
Explained fitted variation (cumulative)	35.130	49.350	60.240	68.160
(b) Variables	Explains %	Contribution %	pseudo-F	P
Salinity	11.3	29.8	15.1	0.002
Region: IE	5.2	13.5	7.2	0.002
Region: DR	3.5	9.1	5.0	0.002
Region: KNR	2.6	6.9	3.9	0.002
Wave exposure: sheltered	2.3	5.9	3.4	0.002
Region: MP	1.9	5.1	3.0	0.002
Region: CH	1.9	5.1	3.0	0.002
Water temperature	1.9	4.9	2.9	0.002
Habitat: block-sediment	1.4	3.7	2.3	0.004
Wave exposure: semi-exposed	1.3	3.5	2.2	0.006
Wave exposure: exposed	1.3	3.5	2.2	0.002
Habitat: block-boulder	1.3	3.4	2.1	0.002
Habitat: rock	1.3	3.4	2.1	0.002
Depth	1.2	3.2	2.0	0.004
*Lessonia*	1.0	2.6	1.6	0.034

*Amphimedon maresi* (Demospongiae), and the bivalves *Aulacomya atra* and *Mytilus chilensis* were most highly correlated with KNR. The barnacle *Balanus laevis* (Cirripedia) and an unidentified orange encrusting bryozoan were highly correlated with IE, while two anthozoans (*Bunodactis octoradiata* and *Anthothoe chilensis*) and two sea urchins (*Austrocidaris canaliculata* and *Loxechinus albus*) were most strongly correlated with MP.

**Fig 6:**
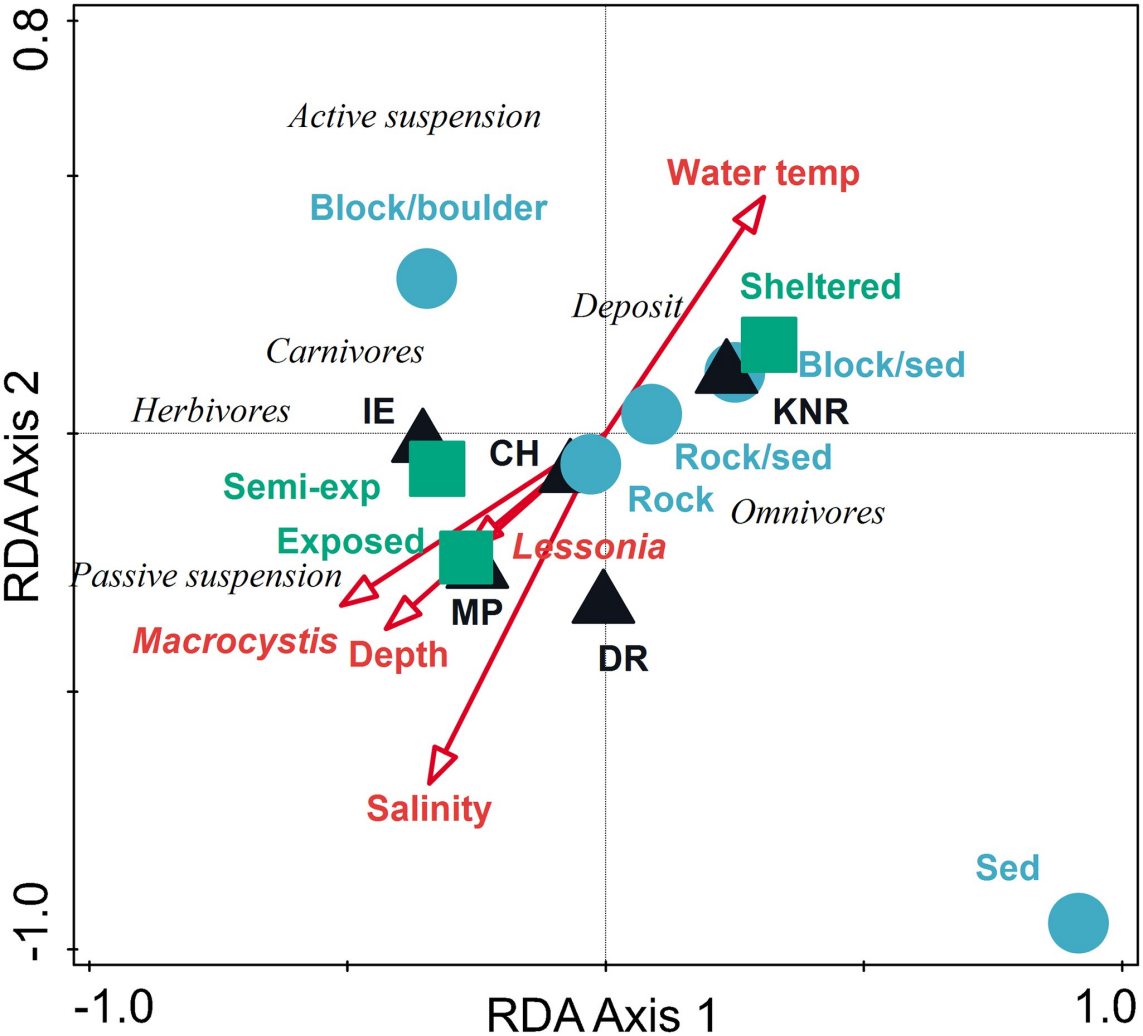
Triplot of results of redundancy analysis on benthic feeding guild abundance by region with biotic and abiotic variables (wave exposure, depth, salinity, temperature, densities of *Macrocystis* and *Lessonia* kelp). Data were centered and ln(x+1) transformed benthic taxa abundance by transect. Biotic and abiotic variables were standardized prior to analysis.

When considering the functional (feeding guild) structure of the benthic assemblages, regions clustered together in ordination space except for KNR, which was well separated from the others (Fig 6). The first two axes of the RDA biplot explained 20.9% of the variance among benthic taxa, with the explanatory variables accounting for 69.2% of total fitted variation (Table 4). Sheltered wave exposure sites explained 8.6% of the total variation and contributed 28.6% to the explained fitted variation, followed by salinity with 12.5% of the fitted variation, and the KNR region (11.2%). Sediment habitat and water temperature each contributed an additional 7.9% of the fitted variation. Sheltered wave exposures and higher water temperatures were correlated with KNR. Deposit feeders and omnivores were most highly correlated with KNR, while herbivorous browsers and passive suspension feeders were most highly correlated with the remaining regions.

**Table 4:** (a) Results of redundancy analysis (RDA) on ln(x+1) transformed benthic feeding guild abundance (num. m^-2^) by transect. (b) Conditional effects of Monte-Carlo permutation results on the RDA.

Statistic	Axis 1	Axis 2	Axis 3	Axis 4
Eigenvalues	0.112	0.097	0.053	0.022
Explained variation (cumulative)	11.160	20.880	26.170	28.370
Pseudo-canonical correlation	0.617	0.673	0.472	0.406
Explained fitted variation (cumulative)	36.980	69.180	86.700	93.980
Variable	Explains %	Contribution %	pseudo-F	P
Wave exposure: Sheltered	8.6	28.6	11.1	0.002
Salinity	3.8	12.5	5.1	0.002
Region: KNR	3.4	11.2	4.7	0.004
Habitat: sediment	2.4	7.9	3.4	0.008
Water temperature	2.4	7.9	3.4	0.008
Region: IE	2.3	7.6	3.4	0.004
Region: DR	1.7	5.5	2.5	0.038

## Discussion

Patagonian kelp forests are some of the least disturbed nearshore habitats globally owing to their remoteness, extreme environmental conditions, and low human population [14, 15]. The extensive kelp forests that dominate the region play a key role in structuring the entire coastal ecosystem of the area [13, 16]. We found diverse assemblages of macroinvertebrates associated with kelp forests across a wide area of southern Patagonia. Taxonomic richness was highest at Isla de los Estados, with the lowest taxonomic richness recorded at the remote Diego Ramírez. Sampling was restricted to kelp forests, so the underlying benthic habitat was predominately rock, although the amount of soft sediment, boulders, and rock did explain some of the variability in assemblage structure. The eastward influence of the Pacific by the Antarctic Circumpolar Current (ACC) and the westward influence of Atlantic waters result in a complex temperature structure through the water column with strong tidal exchange [49, 50]. These strong and varied physical forcing factors result in highly heterogenous marine communities. As a result, biotic and abiotic factors varied greatly among and within regions and only explained a modest amount of the variability in invertebrate assemblage structure. KNR was most distinct from the other regions with a relatively greater abundance of deposit feeders, mainly ophiuroids, but also a large amount of active suspension feeders (e.g., mussels *Aulacomya atra* and *Mytilus chilensis*). Fjord walls act as buffers from physical ocean energy, leaving locations more protected than those exposed to the open ocean. Tidewater and valley glaciers in these fjords create freshwater outflow, resulting in large fluctuations in salinity with high turbidity and nutrients from terrigenous sources and glacial melt, which likely contributed to the prevalence of deposit and active suspension feeders in KNR, mainly in the inner parts of the fjords [18]. Herbivorous browsers and passive suspension feeders were most abundant among the other regions, which did not show large differences in assemblage structure when examined at the feeding guild level.

For many marine invertebrates, dispersal is often strongly influenced by currents and suitable settlement habitat [51, 52]. Isla de los Estados is located 29 km to the east of the southeastern tip of South America and is exposed to influences from the Pacific, Atlantic, and Southern oceans. The westwind drift from the Pacific and Southern oceans carry larvae from western Patagonia and the Antarctic Peninsula to Isla de los Estados, while the Brazil Current moves down the southeast coast of South America before heading east to meet the Malvinas Current [53]. Counter to this, Diego Ramírez is located 105 km west-southwest of Cape Horn, requiring larvae to travel a much greater distance before settling onto suitable substrate for successful recruitment. In addition, the small size and limited habitat of the Diego Ramírez Archipelago makes for a small target for larval settlement. In a complementary study at the same survey sites, Hüne et al. [54] found similar patterns for fishes, with the highest fish species richness and diversity at Isla de los Estados and the lowest at Diego Ramírez.

Dayton [13] hypothesized that the Circumpolar Westwind Drift resulted in low larval availability to the region and documented relatively low densities of the sea urchin *Loxechinus albus* at IE and MP. Our data show similarly low densities of *L*. *albus* to Dayton’s at IE (1.3 ± 1.76 m^-2^) and MP (1.3 ± 1.2), with lower densities of this species at CH (0.6 ± 1.3) and DR (0.7 ± 0.3), and even lower densities at KNR (0.1 ± 0.5). This gradient of densities is consistent with Dayton’s prediction.

We did not observe large aggregations or ‘barrens’ of sea urchins although there are reported to occur further north in the region [13, 55, 56]. This lack of urchin barrens is contrary to many other regions of the world that are experiencing transitions from kelp forests to urchin barrens (e.g., Tasmania, Eastern Australia, Norway, Northern California [36, 56, 57]). In many locations, the loss of fish or invertebrate predators can result in urchin barren formation [58], but this is unlikely to explain the lack of barrens in southern Patagonia as the fish community is made up of smaller species that are not likely to control urchins through predation [54].

What drives the dynamics of these kelp forest and other macroalgal dominated communities is a question that has perplexed scientists for some time [11, 12, 33, 57, 59, 60]. Numerous studies have shown the top-down role that predators play in structuring kelp forests by controlling herbivores, which graze down these kelp forests [23, 58, 61]. However, other studies point to different factors that influence kelp forests dynamics such as physical forcing [59], productivity [62–64], kelp production outweighing herbivore consumption [65], and structural complexity in habitat [60], to name a few. A better understanding of the kelp forests of southern Patagonia may help to tease out some of the drivers of these ecosystems owing to the minimal anthropogenic impacts within this region.

The southern tip of South America hosts the confluence of water masses from three great oceans: the Pacific, Atlantic, and Southern. This creates a mix of species with temperate and sub-Antarctic distributions, and a unique area of marine endemism with the southernmost kelp forests in the world and extremely high biodiversity value [7, 66]. Owing to their distribution, these kelp forests, and the assemblages they host are therefore excellent indicators of large-scale anthropogenic-related changes, mainly with regards to global warming (e.g., temperature increases, lower salinity associated with glacial melt, increasing precipitations, increased sedimentation due to glacial melt).

The southern tip of South America is predicted to warm more slowly than other regions of the world, and unlike other kelp forest ecosystems are currently not showing signs of tropicalization [67]. The resolute strength of the Antarctic Circumpolar Current reliably transports cold water from Antarctica to Patagonia, supplying stability to the region’s temperate nearshore systems [13, 49, 68]. The unimpeded force of this current leaves Patagonia and the South Atlantic as some of the least disturbed and most persistent examples of this ecosystem worldwide [13–15]. Our study provides the first broad-scale description of the benthic assemblages associated with kelp forests in this vast and little-studied area and helps to establish baselines for a region that is currently lightly influenced by local anthropogenic factors and less likely to be impacted by climate change when compared with other kelp forests worldwide.

## Supporting information

S1 Table Benthic taxa identified during expeditions to southern Patagonia. (DOCX)
