# Supplementary material for: Patterns and drivers of benthic macroinvertebrate assemblages in the kelp forests of southern Patagonia
Source: PLoS One. 2023 Jan 6;18(1):e0279200. doi: 10.1371/journal.pone.0279200 (PMC9821511; doi:10.1371/journal.pone.0279200)
Supplement: S1 Table — (DOCX) [file pone.0279200.s001.docx]

S1 Table. Benthic taxa identified during expeditions to southern Patagonia.

| **Phylum** | **Class to Infraorder** | **Species** | **Feeding guild** |
| --- | --- | --- | --- |
| Annelida | Polychaeta | *Spiochaetopterus patagonicus* | active suspension feeder |
| Annelida | Polychaeta | *Spirorbis* sp. | active suspension feeder |
| Annelida | Polychaeta | *Apomatus* sp*.* | active suspension feeder |
| Annelida | Polychaeta | *Chaetopterus variopedatus* | active suspension feeder |
| Annelida | Polychaeta | *Eulalia* sp. | carnivorous |
| Annelida | Polychaeta | *Perkinsiana magalhaensis* | active suspension feeder |
| Arthropoda | Cirripedia | *Arossia henryae* | active suspension feeder |
| Arthropoda | Cirripedia | *Austromegabalanus psittacus* | active suspension feeder |
| Arthropoda | Cirripedia | *Balanus laevis* | active suspension feeder |
| Arthropoda | Cirripedia | *Elminius kingii* | active suspension feeder |
| Arthropoda | Cirripedia | *Notobalanus flosculus* | active suspension feeder |
| Arthropoda | Malacostraca | *Cristaserolis convexa* | carnivorous |
| Arthropoda | Malacostraca | *Acanthocyclus albatrossis* | carnivorous |
| Arthropoda | Malacostraca | *Campylonotus vagans* | carnivorous |
| Arthropoda | Malacostraca | *Eurypodius latreillii* | carnivorous |
| Arthropoda | Malacostraca | *Halicarcinus planatus* | carnivorous |
| Arthropoda | Malacostraca | *Lithodes santolla* | carnivorous |
| Arthropoda | Malacostraca | *Munida gregaria* | omnivorous |
| Arthropoda | Malacostraca | *Nauticaris magellanica* | carnivorous |
| Arthropoda | Malacostraca | *Pagurus comptus* | carnivorous |
| Arthropoda | Malacostraca | *Paralomis granulosa* | carnivorous |
| Arthropoda | Malacostraca | *Peltarion spinulosum* | carnivorous |
| Arthropoda | Malacostraca | *Pisoides edwardsii* | carnivorous |
| Arthropoda | Malacostraca | Unidentified crab *(Cyclograpsus*-like*)* | carnivorous |
| Brachiopoda | Rhynchonellata | *Terebratella dorsata* | active suspension feeder |
| Brachiopoda | Rhynchonellata | *Magellania venosa* | active suspension feeder |
| **Phylum** | **Class to Infraorder** | **Species** | **Feeding guild** |
| Chordata | Ascidiacea | *Aplidium "proliferum"* | active suspension feeder |
| Chordata | Ascidiacea | *Aplidium cf. peruvianum* | active suspension feeder |
| Chordata | Ascidiacea | *Aplidium fuegiense* | active suspension feeder |
| Chordata | Ascidiacea | *Aplidium magellanicum* | active suspension feeder |
| Chordata | Ascidiacea | *Cnemidocarpa nordenskjöldi* | active suspension feeder |
| Chordata | Ascidiacea | *Cnemidocarpa ohlini* | active suspension feeder |
| Chordata | Ascidiacea | *Cnemidocarpa verrucosa* | active suspension feeder |
| Chordata | Ascidiacea | *Corella eumyota* | active suspension feeder |
| Chordata | Ascidiacea | *Didemnum studeri* | active suspension feeder |
| Chordata | Ascidiacea | *Distaplia colligans* | active suspension feeder |
| Chordata | Ascidiacea | *Morchellium giardi* | active suspension feeder |
| Chordata | Ascidiacea | *Paramolgula gigantea* | active suspension feeder |
| Chordata | Ascidiacea | *Polysyncraton trivolutum (Didemnum* white greyish) | active suspension feeder |
| Chordata | Ascidiacea | *Polyzoa opuntia* | active suspension feeder |
| Chordata | Ascidiacea | *Pyura cf. chilensis* | active suspension feeder |
| Chordata | Ascidiacea | *Pyura legumen* | active suspension feeder |
| Chordata | Ascidiacea | *Sycozoa gaimardi* | active suspension feeder |
| Chordata | Ascidiacea | *Synoicum georgianum* | active suspension feeder |
| Chordata | Ascidiacea | *Unidentified Didemnidae* | active suspension feeder |
| Cnidaria | Anthozoa | *Acontiaria unidentified* | passive suspension feeder |
| Cnidaria | Anthozoa | *Actinostola chilensis* | passive suspension feeder |
| Cnidaria | Anthozoa | *Actinothoe lobata* | passive suspension feeder |
| Cnidaria | Anthozoa | *Alcyonium yepayek* | passive suspension feeder |
| Phylum | Class to Infraorder | Species | Feeding guild |
| Cnidaria | Anthozoa | *Antholoba achates* | passive suspension feeder |
| Cnidaria | Anthozoa | *Anthothoe chilensis (aff.)* | passive suspension feeder |
| Cnidaria | Anthozoa | *Bolocera* sp. (small) | active suspension feeder |
| Cnidaria | Anthozoa | *Boloceropsis* sp. | passive suspension feeder |
| **Phylum** | **Class to Infraorder** | **Species** | **Feeding guild** |
| Cnidaria | Anthozoa | *Bunodactis octoradiata* | passive suspension feeder |
| Cnidaria | Anthozoa | *Incrustatus comauensis* | passive suspension feeder |
| Cnidaria | Anthozoa | *Isoparactis fabiani* | active suspension feeder |
| Cnidaria | Anthozoa | *Primnoella chilensis* | passive suspension feeder |
| Cnidaria | Hydrozoa | *Grammaria abietina* | passive suspension feeder |
| Cnidaria | Hydrozoa | *Obelia geniculata* | passive suspension feeder |
| Cnidaria | Hydrozoa | *Plumularia setacea* | passive suspension feeder |
| Cnidaria | Hydrozoa | *Sertularella polyzonias* | active suspension feeder |
| Cnidaria | Hydrozoa | *Symplectoscyphus filiformis* | passive suspension feeder |
| Cnidaria | Hydrozoa | *Symplectoscyphus magellanicus* | passive suspension feeder |
| Cnidaria | Hydrozoa | *Symplectoscyphus subdichotomus* | passive suspension feeder |
| Cnidaria | Hydrozoa | *Unidentified hydrozoan* | passive suspension feeder |
| Echinodermata | Asteroidea | *Anasterias antarctica* | carnivorous |
| Echinodermata | Asteroidea | *Asterina fimbriata* | carnivorous |
| Echinodermata | Asteroidea | *Cosmasterias lurida* | carnivorous |
| Echinodermata | Asteroidea | *Cycethra verrucosa* | carnivorous |
| Echinodermata | Asteroidea | *Diplodontias singularis* | carnivorous |
| Echinodermata | Asteroidea | *Ganeria falklandica* | carnivorous |
| Echinodermata | Asteroidea | *Glabraster antarctica* | carnivorous |
| Echinodermata | Asteroidea | *Henricia obesa* | carnivorous |
| Echinodermata | Asteroidea | *Henricia studeri* | carnivorous |
| Echinodermata | Asteroidea | *Labidiaster radiosus* | carnivorous |
| Echinodermata | Asteroidea | *Lophaster stellans* | carnivorous |
| Echinodermata | Asteroidea | *Mimastrella cognata* | carnivorous |
| Echinodermata | Asteroidea | *Odontaster meridionalis* | carnivorous |
| Echinodermata | Asteroidea | *Odontaster penicillatus* | carnivorous |
| Echinodermata | Asteroidea | *Peribolaster folliculatus* | carnivorous |
| Echinodermata | Asteroidea | *Poraniopsis echinaster* | carnivorous |
| Echinodermata | Asteroidea | *Pteraster gibber* | carnivorous |
| **Phylum** | **Class to Infraorder** | **Species** | **Feeding guild** |
| Echinodermata | Asteroidea | *Solaster regularis* | carnivorous |
| Echinodermata | Asteroidea | *Stichaster striatus* | carnivorous |
| Echinodermata | Asteroidea | Unidentified Long legged spiky star | carnivorous |
| Echinodermata | Asteroidea | Unidentified Six arm fat star | carnivorous |
| Echinodermata | Echinoidea | *Arbacia dufresnii* | herbivorous/browser |
| Echinodermata | Echinoidea | *Austrocidaris canaliculata* | carnivorous |
| Echinodermata | Echinoidea | *Loxechinus albus* | herbivorous/browser |
| Echinodermata | Echinoidea | *Pseudechinus magellanicus* | herbivorous/browser |
| Echinodermata | Holoturioidea | *Chiridota pisanii* | deposit feeder |
| Echinodermata | Holoturioidea | *Cladodactyla crocea* var*. croceoides* | passive suspension feeder |
| Echinodermata | Holoturioidea | *Pentactella leonina* | passive suspension feeder |
| Echinodermata | Holoturioidea | *Psolus patagonicus* | passive suspension feeder |
| Echinodermata | Holoturioidea | *Psolus squamatus* | passive suspension feeder |
| Echinodermata | Ophiuroidea | *Ophiactis asperula* | deposit feeder |
| Echinodermata | Ophiuroidea | *Ophiomyxa vivipara* | deposit feeder |
| Echinodermata | Ophiuroidea | *Ophiuroglypha lymani* | deposit feeder |
| Ectoprocta | Gymnolaemata | *Beania magellanica* | active suspension feeder |
| Ectoprocta | Gymnolaemata | *Beania* sp. | active suspension feeder |
| Ectoprocta | Gymnolaemata | *Bugula* sp. | active suspension feeder |
| Ectoprocta | Gymnolaemata | *Bugula* sp. 2 | active suspension feeder |
| Ectoprocta | Gymnolaemata | *Carbasea ovoidea* | active suspension feeder |
| Ectoprocta | Gymnolaemata | *Cellaria malvinensis* | active suspension feeder |
| Ectoprocta | Gymnolaemata | *Membranipora isabelleana* | active suspension feeder |
| Ectoprocta | Gymnolaemata | *Microporella hyadesi* | active suspension feeder |
| Ectoprocta | Gymnolaemata | Unidentified cervicorn big black bryozoan | active suspension feeder |

| **Phylum** | **Class to Infraorder** | **Species** | **Feeding guild** |
| --- | --- | --- | --- |
| Ectoprocta | Gymnolaemata | Unidentified encrusting orange thin bryozoan | active suspension feeder |
| Ectoprocta | Gymnolaemata | Unidentified encrusting thick orange bryozoan | active suspension feeder |
| Ectoprocta | Gymnolaemata | Unidentified encrusting thin bryozoan | herbivorous/browser |
| Ectoprocta | Gymnolaemata | Unidentified white encrusting bryozoan thin | active suspension feeder |
| Ectoprocta | Gymnolaemata | *Schizomavella* sp.? | active suspension feeder |
| Ectoprocta | Gymnolaemata | *Scrupocellaria* sp. | active suspension feeder |
| Ectoprocta | Stenolaemata | *Disporella* sp. | active suspension feeder |
| Ectoprocta | Stenolaemata | *Entalophora* sp. | active suspension feeder |
| Ectoprocta | Stenolaemata | *Crisia* sp. | active suspension feeder |
| Mollusca | Bivalvia | *Aulacomya atra* | active suspension feeder |
| Mollusca | Bivalvia | *Gaimardia trapesina* | active suspension feeder |
| Mollusca | Bivalvia | *Mytilus chilensis* | active suspension feeder |
| Mollusca | Bivalvia | *Tawera elliptica* | active suspension feeder |
| Mollusca | Bivalvia | *Zygochlamys patagonica* | active suspension feeder |
| Mollusca | Cephalopoda | *Robsonella fontaniana* | carnivorous |
| Mollusca | Gastropoda | *Acanthina monodon* | carnivorous |
| Mollusca | Gastropoda | *Adelomelon ancilla* | carnivorous |
| Mollusca | Gastropoda | *Argobuccinum pustulosum* | carnivorous |
| Mollusca | Gastropoda | *Berthella platei* | omnivorous |
| Mollusca | Gastropoda | *Cadlina sparsa* | carnivorous |
| Mollusca | Gastropoda | *Concholepas concholepas* | carnivorous |
| Mollusca | Gastropoda | *Crepidula dilatata* | herbivorous/browser |
| Mollusca | Gastropoda | *Diaulula hispida* | carnivorous |
| Mollusca | Gastropoda | *Diaulula punctuolata* | carnivorous |
| Mollusca | Gastropoda | *Doris fontainii* | carnivorous |
| **Phylum** | **Class to Infraorder** | **Species** | **Feeding guild** |
| Mollusca | Gastropoda | *Falsilunatia patagonica* | deposit feeder |
| Mollusca | Gastropoda | *Fissurella picta + oriens* | herbivorous/browser |
| Mollusca | Gastropoda | *Fissurellidea patagonica* | herbivorous/browser |
| Mollusca | Gastropoda | *Fusitriton magellanicus* | carnivorous |
| Mollusca | Gastropoda | *Gargamella immaculata* | carnivorous |
| Mollusca | Gastropoda | *Holoplocamus papposus* | carnivorous |
| Mollusca | Gastropoda | *Itaxia falklandica* | carnivorous |
| Mollusca | Gastropoda | *Lamellaria* sp. | carnivorous |
| Mollusca | Gastropoda | *Margarella violacea* | herbivorous/browser |
| Mollusca | Gastropoda | *Nacella flammea* | herbivorous/browser |
| Mollusca | Gastropoda | *Nacella magellanica* | herbivorous/browser |
| Mollusca | Gastropoda | *Nacella mytilina* | herbivorous/browser |
| Mollusca | Gastropoda | *Pareuthria fuscata* | carnivorous |
| Mollusca | Gastropoda | *Phyllidia* sp. | carnivorous |
| Mollusca | Gastropoda | *Scissurella clathrata* | herbivorous/browser |
| Mollusca | Gastropoda | *Tegula atra* | herbivorous/browser |
| Mollusca | Gastropoda | *Thecacera darwini* | carnivorous |
| Mollusca | Gastropoda | *Tritonia challengeriana* | carnivorous |
| Mollusca | Gastropoda | *Trophon geversianus* | carnivorous |
| Mollusca | Gastropoda | *Trophon plicatus* | carnivorous |
| Mollusca | Gastropoda | *Tyrinna delicata* | carnivorous |
| Mollusca | Gastropoda | *Xymenopsis muriciformis* | carnivorous |
| Mollusca | Polyplacophora | *Nuttallochiton martiali* | herbivorous/browser |
| Mollusca | Polyplacophora | *Tonicia atrata+calbucensis+chilensis+lebruni+smithii* | herbivorous/browser |
| Mollusca | Polyplacophora | *Callochiton puniceus* | herbivorous/browser |
| Mollusca | Polyplacophora | *Chiton boweni* | herbivorous/browser |
| Mollusca | Polyplacophora | *Plaxiphora aurata* | herbivorous/browser |
| **Phylum** | **Class to Infraorder** | **Species** | **Feeding guild** |
| Nemertea | Pilidiophora | *Baseodiscus aureus* | carnivorous |
| Nemertea | Pilidiophora | *Unidentified Nemertea* | deposit feeder |
| Porifera | Calcarea | *Clathrina ramosa* | active suspension feeder |
| Porifera | Calcarea | *Leucettusa nuda* | active suspension feeder |
| Porifera | Calcarea | *Sycon* spp. | active suspension feeder |
| Porifera | Demospongiae | *Amphimedon maresi* | active suspension feeder |
| Porifera | Demospongiae | *Biemna chilensis* | active suspension feeder |
| Porifera | Demospongiae | *Clathria mytilifila* | active suspension feeder |
| Porifera | Demospongiae | *Clathria rosetafiordica* | active suspension feeder |
| Porifera | Demospongiae | *Clathrina fjordica* | active suspension feeder |
| Porifera | Demospongiae | *Cliona chilensis* | active suspension feeder |
| Porifera | Demospongiae | *Haliclona caduca* | active suspension feeder |
| Porifera | Demospongiae | *Haliclona cf. porcelana* | active suspension feeder |
| Porifera | Demospongiae | *Hemimycale* sp.1 | active suspension feeder |
| Porifera | Demospongiae | *Hemimycale* sp.2 | active suspension feeder |
| Porifera | Demospongiae | *Hymenancora* sp. | active suspension feeder |
| Porifera | Demospongiae | *Mycale magellanica* | active suspension feeder |
| Porifera | Demospongiae | *Oceanapia spinisphaera* | active suspension feeder |
| Porifera | Demospongiae | *Phorbas ferrugineus* | active suspension feeder |
| Porifera | Demospongiae | *Polymastia* sp. | active suspension feeder |
| Porifera | Demospongiae | *Scopalina* sp. | active suspension feeder |
| Porifera | Demospongiae | Unidentified Chondrillidae | active suspension feeder |
| Porifera | Demospongiae | Unidentified Encrusting red sponge | active suspension feeder |
| Porifera | Demospongiae | Unidentified grey sponge massive | active suspension feeder |
| Porifera | Demospongiae | Unidentified orange encrusting sponge | active suspension feeder |
| Porifera | Demospongiae | Unidentified yellow sponge *Hexadella pruvoti-*like | active suspension feeder |
